# Electromyography-Driven Exergaming in Wheelchairs on a Mobile Platform: Bench and Pilot Testing of the WOW-Mobile Fitness System

**DOI:** 10.2196/16054

**Published:** 2021-01-19

**Authors:** James Enciso, Dhruval Variya, James Sunthonlap, Terrence Sarmiento, Ka Mun Lee, James Velasco, Roxanna N Pebdani, Ray D de Leon, Christine Dy, Stefan Keslacy, Deborah Soonmee Won

**Affiliations:** 1 Department of Electrical and Computer Engineering California State University, Los Angeles Los Angeles, CA United States; 2 Rufus Labs Los Angeles, CA United States; 3 Siemens PLM Software Los Angeles, CA United States; 4 University of Sydney Sydney Australia; 5 School of Kinesiology and Nutritional Science California State University, Los Angeles Los Angeles, CA United States

**Keywords:** exergaming, gamercising, mobile health, wheelchair exercises, wireless electromyography, mobile phone

## Abstract

**Background:**

Implementing exercises in the form of video games, otherwise known as exergaming, has gained recent attention as a way to combat health issues resulting from sedentary lifestyles. However, these exergaming apps have not been developed for exercises that can be performed in wheelchairs, and they tend to rely on whole-body movements.

**Objective:**

This study aims to develop a mobile phone app that implements electromyography (EMG)-driven exergaming, to test the feasibility of using this app to enable people in wheelchairs to perform exergames independently and flexibly in their own home, and to assess the perceived usefulness and usability of this mobile health system.

**Methods:**

We developed an Android mobile phone app (Workout on Wheels, WOW-Mobile) that senses upper limb muscle activity (EMG) from wireless body-worn sensors to drive 3 different video games that implement upper limb exercises designed for people in wheelchairs. Cloud server recordings of EMG enabled long-term monitoring and feedback as well as multiplayer gaming. Bench testing of data transmission and power consumption were tested. Pilot testing was conducted on 4 individuals with spinal cord injury. Each had a WOW-Mobile system at home for 8 weeks. We measured the minutes for which the app was used and the exergames were played, and we integrated EMG as a measure of energy expended. We also conducted a perceived usefulness and usability questionnaire.

**Results:**

Bench test results revealed that the app meets performance specifications to enable real-time gaming, cloud storage of data, and live cloud server transmission for multiplayer gaming. The EMG sampling rate of 64 samples per second, in combination with zero-loss data communication with the cloud server within a 10-m range, provided seamless control over the app exergames and allowed for offline data analysis. Each participant successfully used the WOW-Mobile system at home for 8 weeks, using the app for an average of 146 (range 89-267) minutes per week with the system, actively exergaming for an average of 53% of that time (39%-59%). Energy expenditure, as measured by integrated EMG, was found to be directly proportional to the time spent on the app (Pearson correlation coefficient, r=0.57-0.86, depending on the game). Of the 4 participants, 2 did not exercise regularly before the study; these 2 participants increased from reportedly exercising close to 0 minutes per week to exergaming 58 and 158 minutes on average using the WOW-Mobile fitness system. The perceived usefulness of WOW-Mobile in motivating participants to exercise averaged 4.5 on a 5-point Likert scale and averaged 5 for the 3 participants with thoracic level injuries. The mean overall ease of use score was 4.25 out of 5.

**Conclusions:**

Mobile app exergames driven by EMG have promising potential for encouraging and facilitating fitness for individuals in wheelchairs who have maintained arm and hand mobility.

## Introduction

Individuals with paraplegia are at a greater risk for many secondary health problems associated with sedentary behavior [1-3]. The benefits of physical exercise on the health and quality of life of people with disabilities have been reported [4-6]. Dishearteningly, individuals with impaired mobility face substantial barriers to exercise, such as difficulty in accessing exercise programs and facilities, which contribute to an overall reduction in participation in physical activity [7-9]. With the known benefits that physical exercise has on health, digital sensor-driven technology is being considered as an approach to make exercise more accessible and entertaining [10-14]. Exercising in the process of achieving the objectives of a digital video game is termed exergaming [15,16]. Reportedly, 61% of internet-based exercise interventions lead to significant gains in physical activity [17]. Some researchers have gamified exercises to encourage more active lifestyles [16,18,19]. A recent review paper examined studies showing the beneficial health effect of exergaming and pointed to the ripe opportunity to apply exergaming to the health issues that individuals with neurological disabilities face [14]. At its inception, *exergaming* was popularized as arcade games or console games and has more recently been implemented as desktop and web apps [20-25]. Now, exergaming is beginning to appear on mobile platforms (namely, smartphones and tablets) [16,26,27], which could help in overcoming transportation challenges and inaccessible gym environments for people using wheelchairs. However, among those that have been implemented, there are none to the authors’ knowledge that are tailored to exergaming in wheelchairs.

We have developed a mobile app that communicates with body-worn sensors that monitor physical activity and feed electromyography (EMG) input into a mobile app game engine that gamifies exercises designed to be carried out independently by individuals in wheelchairs. The current recommendations for exercise regimens for individuals in wheelchairs holistically combine cardiovascular conditioning and strength training [28]. Evidence of the need for building muscle strength to prevent overuse injury and pain and to enable individuals in wheelchairs to sustain sufficient exercise on a weekly routine basis has led to the development of exercise interventions, such as circuit resistance training (CRT), which include muscle strengthening [28,29]. CRT entails interspersing arm resistance strength exercises (such as weight lifting) with high-speed cardiovascular exercise (such as arm cranking) and incomplete recovery periods during which the heart rate was still sustained well above the resting heart rate. Our app implements these exercises in the form of 3 different games that allow the user to engage in a combination of resistance and aerobic conditioning activities developed to be used in a wheelchair.

Many of the existing fitness apps rely on heart rate and accelerometers. The well-known exergames (eg, Dance Dance Revolution and balance board–centered Wii Fit) rely on step detection or lower limb mobility for an effective workout [12,21-23,30,31]. These systems use pressure sensors to detect body weight or accelerometers to detect ballistic or discrete movements. Accelerometers have sufficient resolution to detect steps and therefore have been relatively effective in fitness apps to date when the physical activity being tracked involves moving the whole body mass. In contrast, detecting muscle activity provides a real-time measurement of continuous changes in physical exertion, that is, by sensing muscle activity via EMG, we would be able to detect the continuous intensity of each muscle contraction rather than only binary detection of a movement. This is especially important in the case of individuals with paraplegia, where the movements involve only the upper body and not their whole body and where strength training entails isometric contractions rather than binary actions (eg, steps, cycles). Commercial wireless EMG sensors are beginning to be used with mobile apps for applications such as monitoring driving and monitoring cadence while biking [32,33]. Our app senses EMG to measure the amount of muscle activity continuously used. Therefore, our app can sense the strength of isometric contractions during muscle strengthening exercises as well as how hard a wheelchair push was during spinning exercises.

The limitation of the existing technology to facilitate and encourage exercise for individuals with lower limb mobility impairment is the lack of a combination of providing exergaming on a mobile platform and tailoring games toward exercises that can be performed in wheelchairs; of particular need for such exergames is the ability to track isometric contractions through EMG sensing. Our objective was to design, implement, and test the feasibility of an EMG-based mobile exergaming app for individuals in wheelchairs. Our mobile app is distinct from other fitness apps in a few key ways. First, the app gamifies exercises that can be performed in a wheelchair on a mobile platform while monitoring effort and providing feedback, thereby making exercises entertaining and accessible. Second, the selection of the exercises was informed by research on the fitness needs of individuals who use wheelchairs as a primary mode of transportation. In particular, the exercises were specifically selected so that individuals in wheelchairs could exercise independently without relying on access to adapted equipment or specialists or physical therapists for their daily physical exercise. Third, the games were driven by EMG, which enables higher resolution and continuous readings of physical exertion from upper limb movements.

The purpose of this study, therefore, is to describe the development and feasibility of a mobile phone app that implements EMG-driven exergaming to encourage and enhance exercise for individuals who use wheelchairs. In this work, we assess the perceived usefulness and usability of this mobile health system by asking the following research questions:

1. Does this mobile fitness app enable individuals who use wheelchairs to increase their level of physical activity?
2. Does use of this mobile fitness app allow individuals who use wheelchairs to reach their self-reported peak fitness levels?
3. Does this mobile fitness app improve self-reported motivation to exercise?
4. Does this mobile fitness app enhance the effectiveness of a workout session for individuals who use wheelchairs?
5. Does this mobile fitness app allow the user to track their progress?
6. How do users perceive the ease of use of this app?

These questions pertain not to a generic mobile fitness app but to one specifically designed to enable people in wheelchairs to perform exergames, used in CRT, independently and flexibly in their own home. As such, the Methods section describes the implementation of our Workout on Wheels (WOW) mobile fitness app.

## Methods

### Mobile Fitness App Concepts

We designed a mobile fitness app, called Workout on Wheels—Mobile (or WOW-Mobile), to encourage and facilitate exercises at home for individuals who have lower mobility impairment and use wheelchairs for ambulation. Table 1 provides an overview of the features or enabling technology that we incorporated into our app design to achieve specific objectives. Ultimately, the goal of the overall design of the app is to help app users achieve greater fitness levels than without the app.

The hardware system and architecture are described elsewhere [34]. Here, we describe the implementation of our design and show the feasibility of achieving these design objectives through the integration of EMG sensing with our mobile fitness app. All code was written in Java using the Android Studio IDE, and the mobile app was installed and tested on the Samsung Galaxy J3.

**Table 1:** Features implemented in the Workout on Wheels-Mobile app and the corresponding enabling design.

Objective	Enabling design or technology
Goal-oriented exercise	Exergaming
Holistic exercise workout in wheelchairs	Spinning, boxing, and arm resistance games
Increase in fitness levels	Electromyography-driven game engines (game performance correlates with effort level)
Fitness tracking	Calories metric, trends page
Encouragement	Audio feedback, text pop-ups
Independence, flexibility	Wireless sensing, mobile platform
Competition	Multiplayer gaming
Socialization	Leaderboard

### Sensor Validation and App Bench Testing

Wireless EMG sensors (Flexdot, Dynofit Inc) were used to drive the exergames and provide feedback on estimated energy expenditure (Multimedia Appendix 1). In addition to the 3 exergames described in the following sections, users also had access to a monitoring activity, which simply displays the signals obtained from the wearable sensors. To validate the EMG acquisition and sensing, we collected surface EMG readings from the bicep bracii muscle from 2 brands of sensors: the Flexdot and the Trigno (Delsys, Inc), a popular high-end commercial wireless EMG sensor and data acquisition system. The mobile fitness system was tested in our research laboratory for power consumption and data transmission performance using Android Studio Profiler. The results from this testing are presented in a later section (*WOW-Mobile Validation and Bench Testing*).

### EMG-Driven Exergame and Monitoring Concepts

We created 3 video games within our mobile phone app (Multimedia Appendices 2-4) to implement corresponding exercises that were developed by coauthors from the School of Kinesiology as part of a circuit training regimen [35]. de Leon established a mobility center on our campus that provides individuals in our community with mobility impairment because of spinal cord injury (SCI) and other causes with very low-cost physical therapy. Other coauthors served as trainers in the clinic and led the development of the exercise protocol on which the exergames were based. The training circuit was designed to help people with SCI achieve recommended cardiorespiratory intensity levels and provide strengthening and endurance to help prevent repetitive use injuries [28,36]. A brief description of the gamified exercises is provided in Table 2. Each game provided an entertaining objective that users could focus on and help them exercise at appropriate intensity levels without focusing on the exercise themselves. It also provided feedback to the user to encourage gains in strength, endurance, and cardiorespiratory fitness in the form of game performance metrics. Audio-visual feedback was incorporated to encourage users to meet the game objectives.

**Table 2:** Conceptual design of Workout on Wheels-Mobile exergames.

Game	Analogous exercise	Exercise objective	Game objective	Feedback provided
Racing	Spinning	High cadence, low resistance	Complete designated number of laps within target time	Time to complete given number of laps. HR^a^, EMG^b^ level, and METs^c^. Audio of car engine; visual of car speed based on EMG level.
High striker	Resistance armbands	Isoinertial resistance (via shoulder press, chest fly, bicep curls)	Raise bar level to upper target with EMG	Number of flexions detected; number of hits of upper target. Audio (bell) when upper target reached. Visual of bar height based on the EMG level. Max EMG reached, HR, and METs.
Boxing	Ball exchange	Maintain HR with a high-cadence, low-resistance exercise + adds variety.	Complete 3 rounds of punches	Audio (punch sound) and visual (stars) with each detected punch.

^a^HR: heart rate.

^b^EMG: electromyography.

^c^MET: metabolic equivalent.

### Calibration and Goals

Thresholds must be set before playing an exergame to appropriately calibrate each game performance with the user’s effort level. A calibration activity was developed, which guides the user through 3 maximum voluntary contractions (MVCs) of the selected muscle (tested separately for the bicep, tricep, anterior deltoid, and posterior deltoid). The average EMG level over a 3-second period during the MVC was used as the 100% effort level. We measured the MVC during orientation in a laboratory setting. EMG thresholds were required for the detection of each muscle contraction in the boxing and high striker game and to calibrate the car’s speed in the racing game.

**Figure 1:**
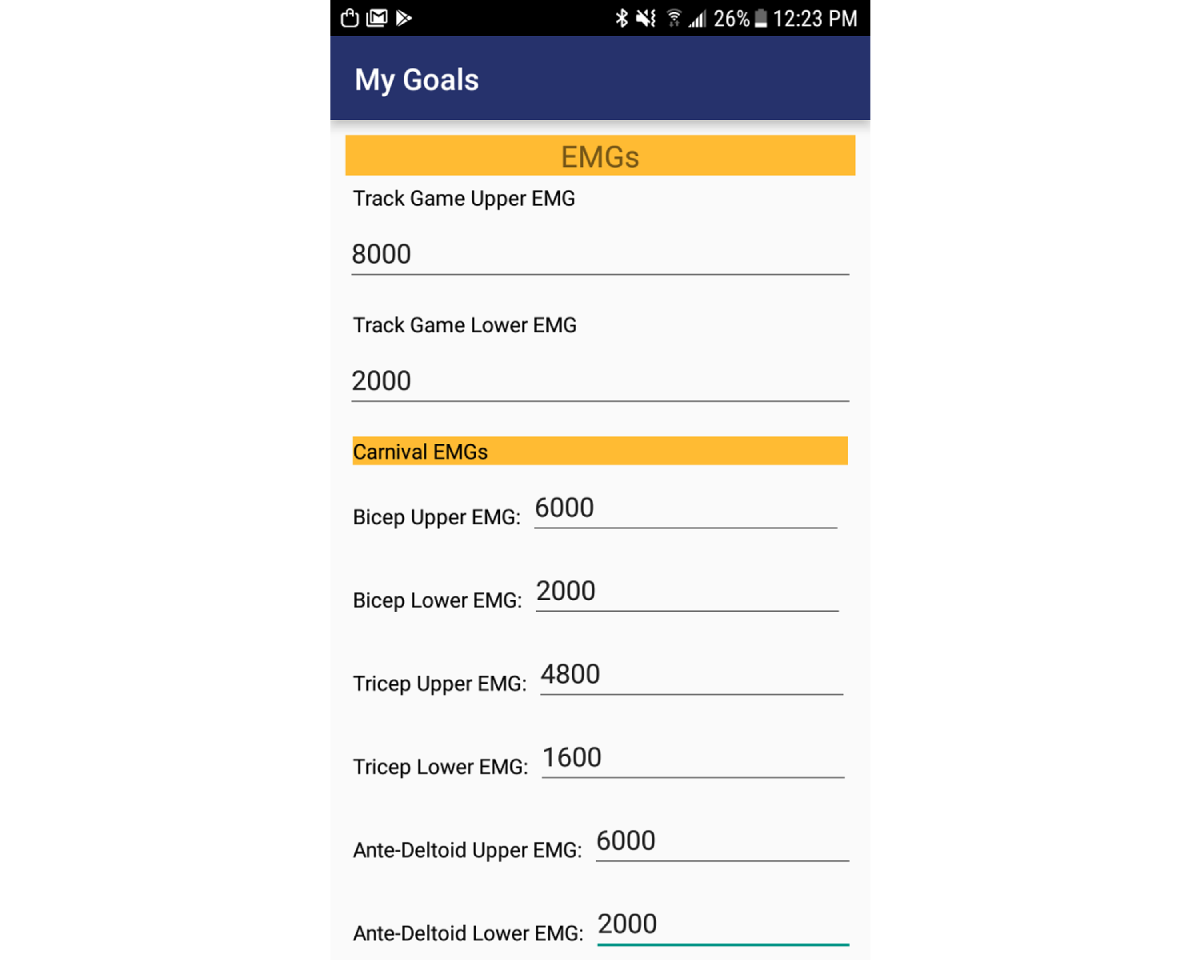
Screenshot of the My Goals page, which stores user-defined electromyography thresholds for each game. EMG: electromyography.

A *MyGoals* activity (Figure 1) was also created to allow the user to save their thresholds so that it does not need to be reentered for each session; rather, the thresholds are fetched for the appropriate game at the start of each session. MVC was measured at baseline testing using the *Calibration* activity. For the high striker game, the upper and lower thresholds were set to 90% and 20%, respectively, of the baseline MVC; for the boxing game, the upper and lower thresholds were set to 80% and 30%, respectively, of MVC. For the racing game, the upper threshold was set to 100% of the MVC, and the lower threshold was set to the empirically determined noise floor.

#### High Striker: Arm Resistance Band Game Design and Implementation

**Figure 2:**
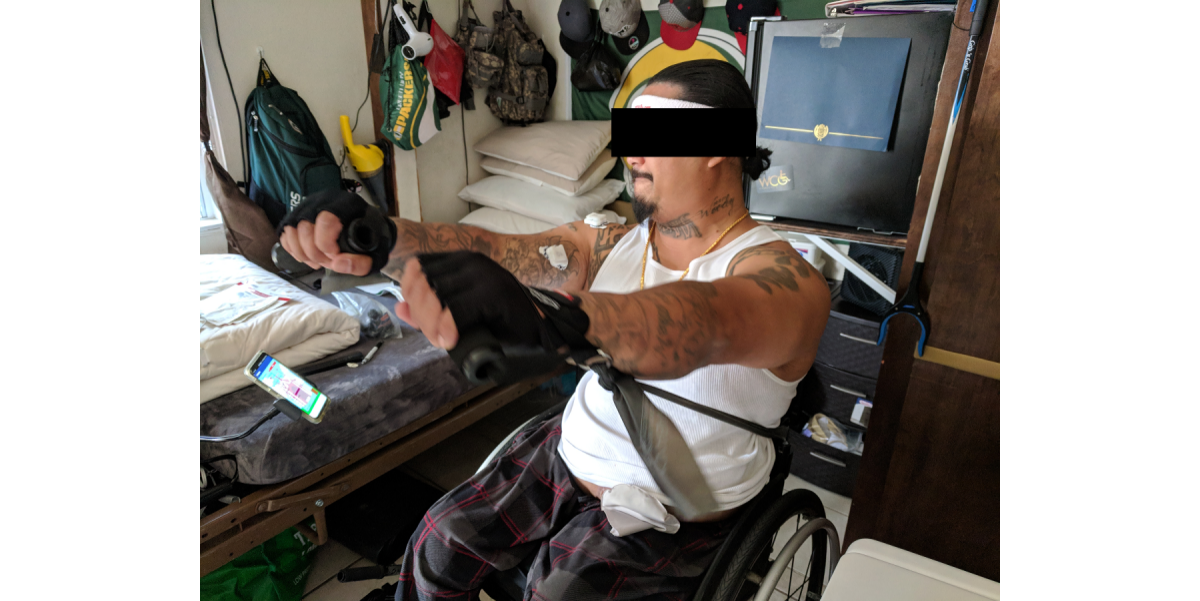
User carrying out chest press exercise to play the high striker exergame.

**Figure 3:**
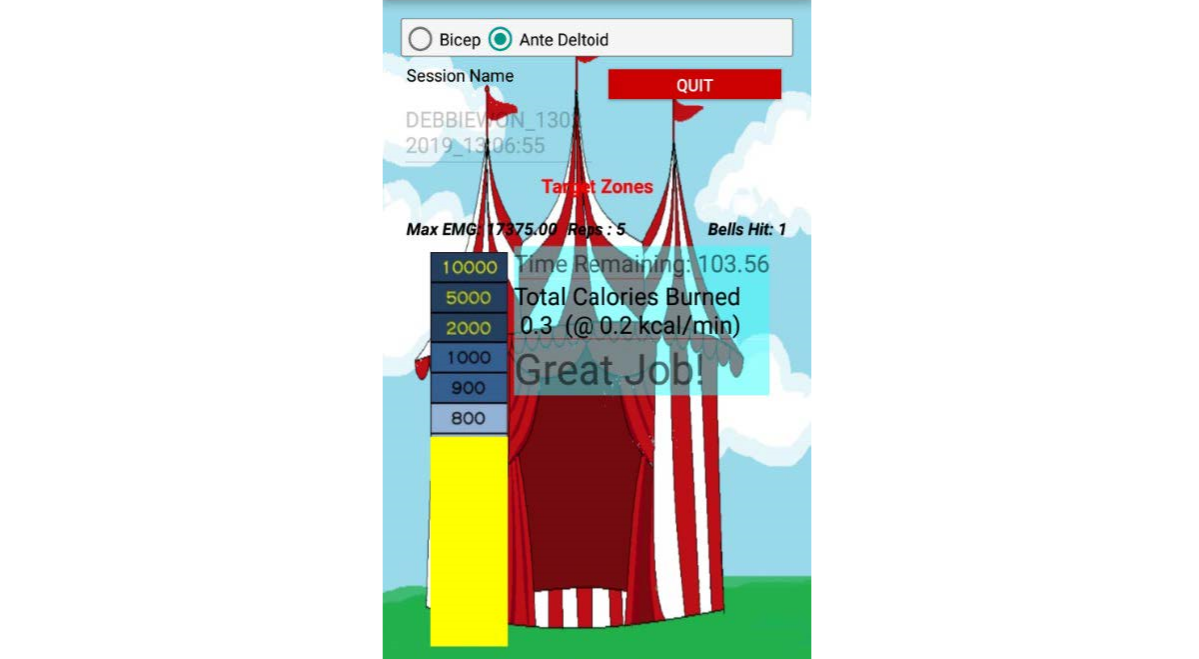
Screenshots showing the app interface for the high striker arm resistance band exergame. User selects which muscle to monitor. The ratio of the yellow bar height to the scaled background bar is equal to the iEMG: MVC level. (a) Interface during the middle of the game. Feedback also includes encouraging text, number of reps, and number of bells hit. (b) Interface at the end of the game, providing summary statistics and a prize based on the number of bells. iEMG: integrated electromyography; MVC: maximum voluntary contraction.

The high striker game replicated the game typically found at carnivals (Multimedia Appendix 2). The player hits one end of a lever to launch a puck up a graduated column. The greater the force the player uses, the higher the puck climbs up the height of the column. The player wins if the puck reaches the top of the column and strikes a bell. We implemented resistance arm band exercises as a game based on the high striker (Figure 2). The column is represented by a bar whose height is proportional to the integrated EMG level over a given contraction (Figure 3). The user selects which muscle’s EMG should drive the bar’s height according to the exercise they plan to perform and on which they are currently focusing (eg, biceps for the bicep curls or anterior deltoids for the shoulder press and chest fly). As the user performs each contraction, the number of contractions (or *hits*) detected is incremented, and the number of times the maximum target (or *bell*) was hit is incremented. The user interface also includes encouraging text pop-ups, upbeat background music, and the bell audio clip each time the bar reaches the maximum.

#### Boxing: Exchange Game Design and Implementation

**Figure 4:**
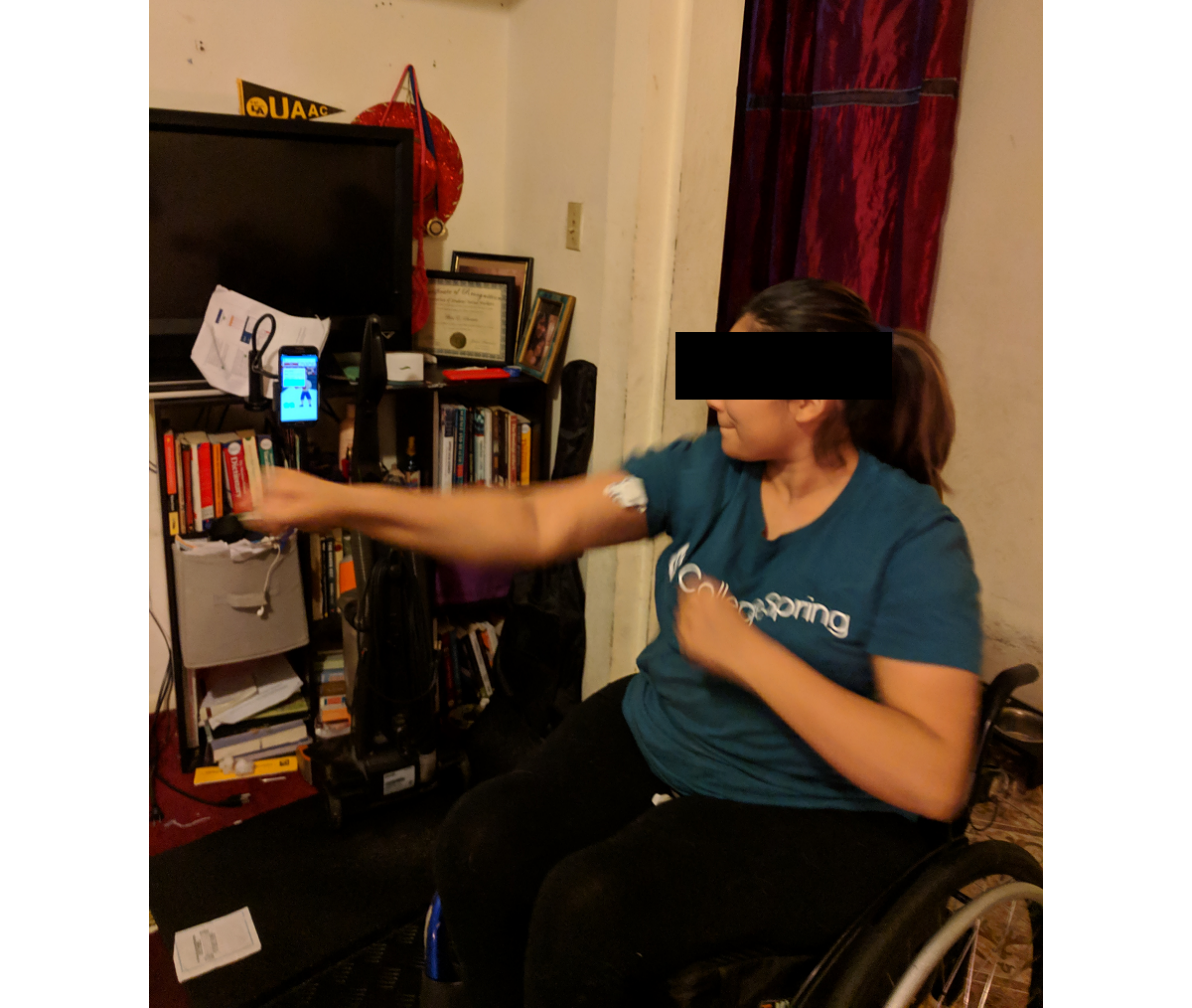
Participant plays the boxing exergame. The EMG sensor can be seen on the right bicep; phone is suspended by phone holder so that the user can monitor progress while playing. EMG: electromyography.

**Figure 5:**
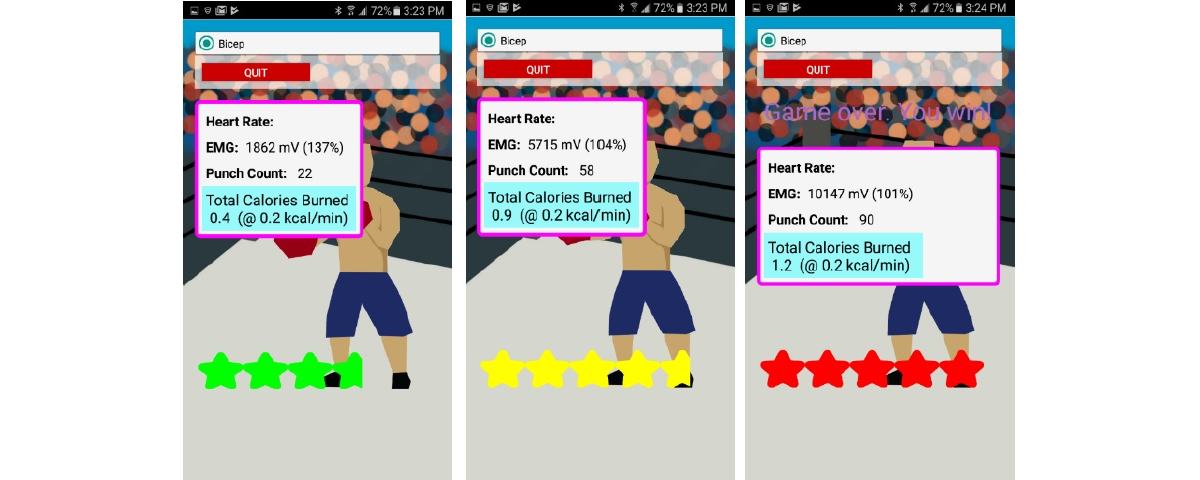
Sequence of screenshots during the boxing game. Each round gets progressively more difficult (the threshold for a punch being detected increases).

The workout developed for this research project included a second high cadence, low-resistance exercise to add variety to the cardiorespiratory exercise, maintain heart rate between the other exercises, and reduce the risk of overuse injury. This exercise was implemented as a boxing game (Figure 4, Multimedia Appendix 4). Players need to complete 30 punches to advance to the next round; there are 3 rounds (Figure 5). To progressively increase the workout intensity, the threshold that defined what constituted a punch increased with each round.

#### Car Racing: Cardio-Spinning Game Design and Implementation

**Figure 6:**
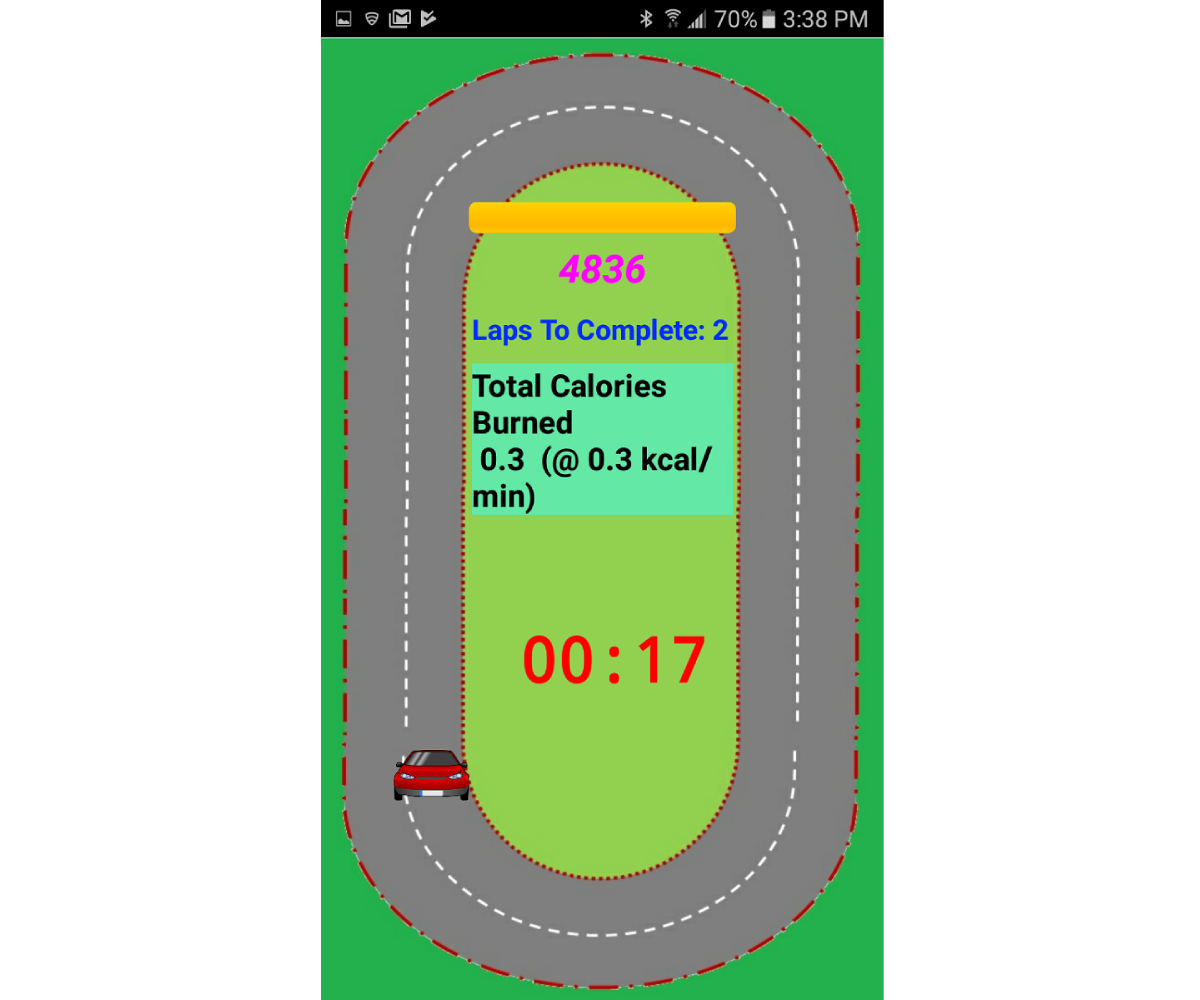
Single-player track game: Angular speed of the car is proportional to the EMG (level for a punch being detected increases). EMG: electromyography.

The spinning exercise was implemented as a car racing game (Figure 6, Multimedia Appendix 3). The angle of the elliptical path around the track increased in proportion with the effort level, whereas the user spun on a stationary roller (Invictus Active Trainer). The effort level was computed as the ratio of the EMG amplitude to the MVC for the given muscle. The metrics displayed to the user during the game included the elapsed time, number of laps to complete, total calories burned, and the current METs. The sound of an engine running would play as background audio throughout the game, whereas an audio clip of *One final lap!* would play as the lap counter decreased to 1 to encourage the user.

We also implemented a multiplayer gaming feature that allows users to select a previous session to be played back as a ghost player against which to race. This feature is outside the scope of this paper and is described in a separate paper.

### Pilot and Feasibility Testing

In total, 4 individuals with incomplete SCI took home and used our WOW-Mobile system for 8 weeks. The California State University, Los Angeles institutional review board approved all study procedures (#18–273). Participant demographics are listed in Table 3. Of 4 participants, 1 (25%) already had an adapted gym at their home and exercised regularly before participating in the study, another had access to adapted exercise equipment in the apartment building, and the other 2 did not have access to a gym and did not exercise regularly before participating in the study. None of the participants had prior experience with exergaming.

**Table 3:** Demographics of study participants.

Subject ID	Age	Gender	Ethnicity	Injury level	Years since injury	Regular exercise at baseline?	Exercise in home?
S1	28	Female	Hispanic	T12	6	No	No
S2	40	Male	Hispanic	T12	13	Yes	Yes
S3	52	Male	Hispanic	T6	10	No	No
S4	38	Male	African American	C7	17	Yes	No

Before beginning the 8 weeks, each participant came to our campus for orientation to the mobile fitness system in a controlled laboratory setting. Physical trainers from the Kinesiology department gave instructions on how to carry out the exercises at home, and they, along with engineering research students who developed the app, guided the participants through a practice session of placing sensors, positioning the armbands, and carrying out the exergames on the mobile app. These physical trainers and engineering students then went to the participants’ homes to set up a stationary spinning device and provided a mobile phone with WOW-Mobile installed, sensors, electrodes, spare batteries, and resistance armbands (TheraBand) and guided the participant one more time through the mobile fitness workout. A workout frequency of 3 times a week for 45 minutes each time was recommended to the participants. The messaging mobile app WhatsApp (Facebook, Inc) was installed on the participants’ phones; a chat room including the participants, engineers, and trainers was created. Participants were instructed to provide feedback regarding the WOW-Mobile app and were encouraged to message the group any time they had issues or questions regarding the app. The participants were visited once at 4 weeks to replenish supplies and check if there were any problems they faced using the app that could better be addressed in person. Participants were paid a weekly US $25 participation stipend for logging into the app at least twice a week, but participants were free to use the app as they chose. This was the same compensation provided for a separate study on gym-based exercise.

Data written to the cloud from each game session were analyzed for number of log-ins, time spent on the app, and actual time spent playing the games. In addition, by analyzing the acquired EMG signals, we also measured the number of detected muscle contractions, the integrated EMG levels (iEMG), and peak EMG levels during each game session using custom-written MATLAB code (Mathworks, Inc). A Likert-scale survey, based on a widely used questionnaire for the *perceived usefulness*, *perceived usability*, and *user acceptance of information technology*, was administered on the web after the 8-week training period.

Before any pilot testing on these 4 participants, 2 other participants were enrolled to conduct feasibility testing. Feasibility testing was conducted to ensure that individuals with moderate and very limited upper mobility would be able to set up and carry out the exergames on their own. These participants helped provide feedback on the app, and several features were modified and some functionality was corrected as a result of their input. Examples include changing the background in the racing game, making threshold adjustments more user-friendly with the calibration feature and saving the user’s default thresholds, and enlarging the control buttons on the screen to make navigating the app more user-friendly.

### Usability of the System

The usability of the system was addressed via a web survey administered through Qualtrics. Participants who had used the WOW-Mobile app were asked 21 questions regarding the usability and usefulness of the app. Of the 21 questions, 18 (86%) were ranked on a Likert scale and 3 were open ended. All 4 WOW-Mobile pilot participants responded to the survey. The mean and SDs were calculated using SPSS Statistics 24 for the quantitative questions, and the qualitative questions are described below. Given that the sample size was 4 and the qualitative responses were very brief, these responses were not analyzed using a qualitative coding method and are presented below.

## Results

### WOW-Mobile Validation and Bench Testing

The performance specifications of the wireless sensors are provided in Table 4. The sampling rate is sufficiently fast to provide what appears to the user to be continuous monitoring of muscle activity, heart rate, and acceleration. All use Bluetooth Low Energy, which provides reliable wireless transmission of the physiological data while optimizing for energy consumption. They are all battery operated, and batteries can either be very easily replaced or have a rechargeable battery.

**Table 4:** Workout on Wheels-Mobile sensor performance specifications.

Characteristics	Electromyography sensor	Alpha 2 heart rate monitor (Mio Global)	Custom accelerometry module
Sampling rate	64 Hz	Continuously	4 Hz
ADC^a^ resolution	15 bits	Unknown (1 BPM^b^)	10 bit
Dynamic range	0-60 V	30-220 BPM	±8G
Dimensions	3.5 cm×3.5 cm×1.2 cm	4.5 cm×3.2 cm×1.5 cm+wrist strap	3.8 cm×5 cm×1.27 cm+wrist strap
Wireless protocol	Bluetooth Low Energy	Bluetooth Low Energy	Bluetooth Low Energy
Battery	3 V 210 mAh Li coin cell	3.7 V 170 mAh Li-Po	Rechargeable 3.7 V 500 mAh Li-Po
Battery life	8 hours of transmission	5 years	5 years

^a^ADC: analog to digital converter.

^b^BPM: beats per minute.

**Figure 7:**
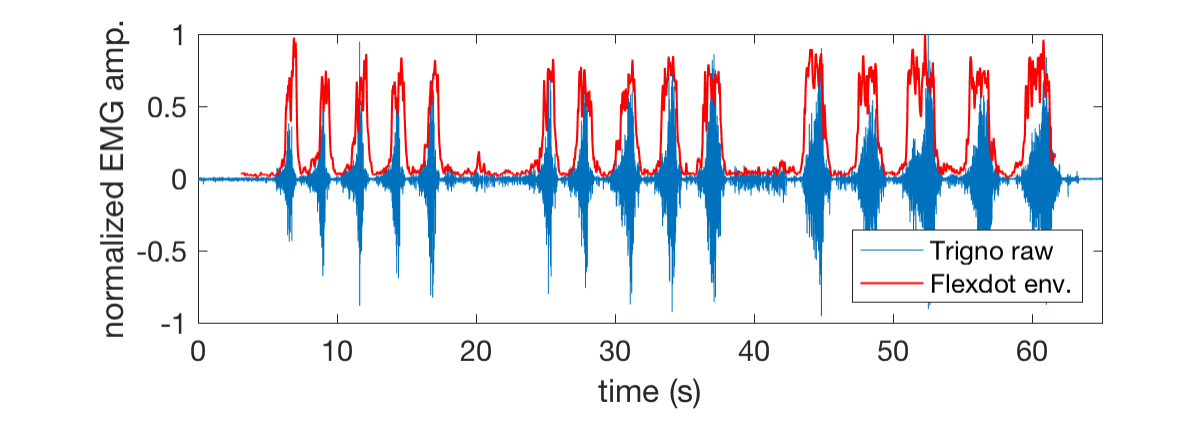
EMG acquired by the Flexdot sensors (red) accurately captured the envelope of the raw EMG activity that was measured by high-end commercial EMG sensors (blue). EMG: electromyography.

The raw EMG from the Trigno during 3 sets of 5 bicep curls is shown in blue in Figure 7. The Flexdot performs on-board signal processing and transmits a 2-pole low-pass filtered EMG envelope, shown in red in Figure 7. The Flexdot envelope can be seen to accurately track the gold standard activity acquired by Trigno. Some differences are expected because of the differences in the position of the sensors. The Flexdot and Trigno were placed adjacent to each other on the same muscle belly. The completely stand-alone wireless nature of the Flexdot and the Bluetooth transmission enable the user to use our app virtually anywhere at any time. Other commercial wireless EMG systems require a base station connected via a USB cable to a computer or otherwise require tethering to a computer.

All data collected from the connected sensors were written to the server at the end of each game session. Data transmission was monitored on Android Studio Profiler, whereas the user wearing the sensors walked gradually away from the phone. The range of transmission for the Bluetooth connection was 10 m inside the building and as far as 100 m in an unobstructed environment. Within the 10-m range, there was zero packet loss. Server upload and download speed were monitored on Speedtest by Ookla and was measured to be 54.8 Mbps and 55.3 Mbps, respectively. Data from a 10-second game with 1 Flexdot connected, for example, require 5 milliseconds on average to write.

### Pilot Study Results

**Figure 8:**
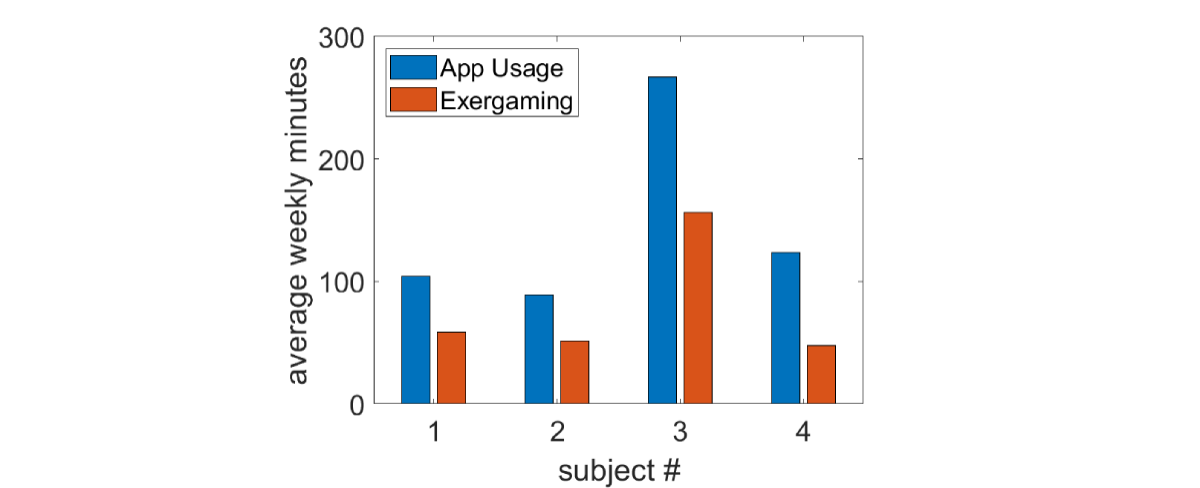
The average number of minutes spent per week using the app and exergaming by each participant.

**Figure 9:**
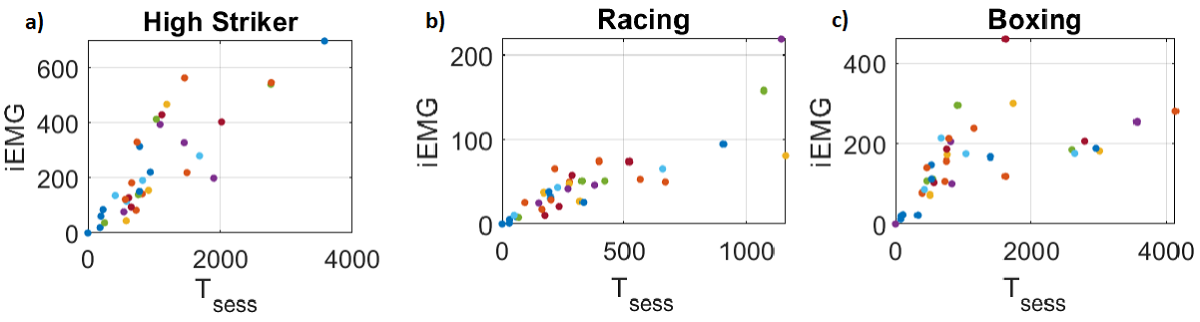
Scatterplots indicating the correlation between time spent on app and energy expenditure, as measured by iEMG. iEMG: integrated electromyography.

A total of 4 participants with varying levels of SCI, whose primary mode of ambulation is by wheelchair, exergamed on a weekly basis for 8 weeks using our WOW-Mobile app. The mean time spent on the app ranged from 89 to 267 minutes per week (Figure 8). The 2 participants who reportedly did not exercise at the start of the study (T12 and T6 injuries) averaged 58 (SD 24) and 157 (SD 61) minutes of exercise per week during the study. The participant with a C7 injury, who had not previously exercised in his home, averaged 48 (SD 14) minutes per week, and the participant who already had an adapted gym in his home averaged 52 (SD 18) minutes per week. From the EMG collected on the cloud server, we measured the total integrated EMG, which is linearly related to energy expenditure [37]. iEMG and inferred energy expenditure increased in proportion to the time spent on the app (Figure 9; r=0.86); that is, the more they used the app, the more energy they expended. In contrast, the maximum EMG level during the sessions, or peak EMG, did not correlate with the time spent on the app (r=0.040), as would be expected, because the peak value is fairly arbitrary—the goals of the games did not encourage them to try to hit their true MVC. Similar results were found for the racing game (r=0.86 for iEMG vs total session time) and boxing (r=0.57).

**Figure 10:**
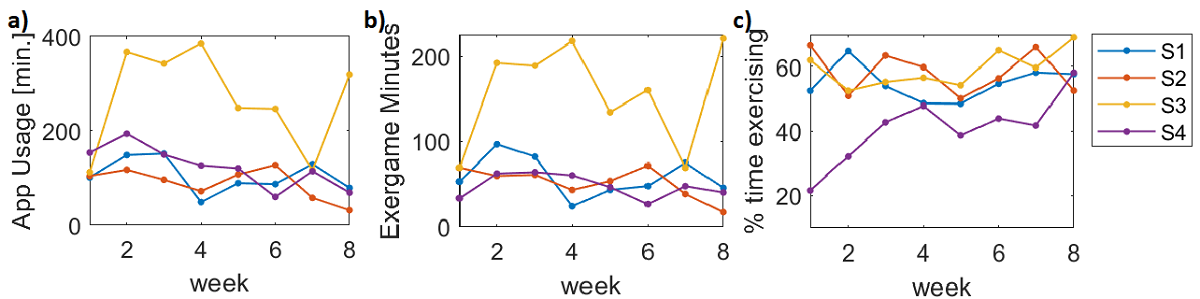
WOW-Mobile app usage over the 8-week period. (a) Total number of minutes spent on the app. (b) Number of minutes spent in exergaming sessions on the app. (c) Percent time on app spent exergaming.

Figure 10 shows that the minutes spent exergaming each week varied from week to week. From the group chat, participants indicated certain weeks that were busier and did not feel able to make more time for exercising. Participant S3 far exceeded other participants in minutes spent exergaming. This participant indicated through the group chat the most interest in the leaderboard and how to improve his rank in the leaderboard. He also expressed hesitation with allowing others to see him while exercising.

The percentage of time logged onto the app that was spent in the exergaming sessions ranged from 48% to 69% for S1, S2, and S3. S4 was the only participant who had hand mobility impairment and had to use his knuckles to tap the screen and had a home care helper to help with snapping electrodes to the sensors. By week 3, his efficiency reached 43%, and by week 8, his efficiency reached 58%.

Over the course of 8 weeks, 234 messages, comprising 20 conversations or *threads*, were sent over the WhatsApp group chat. The 4 participants reported a total of 18 issues and concerns about the WhatsApp group chat, 9 (50%) of which were related to the mobile app itself. These included issues regarding difficulty assigning sensors, games not working because the threshold was not set appropriately, and app crashing when Wi-Fi connectivity was lost, and once because of a billing issue with the cloud service that disabled app use for a day until service was restored. Most of these issues were resolved by week 2. Other nonapp-related issues included running out of the disposable EMG electrodes or batteries or not feeling physically well enough to exercise. There were a couple of conversations consisting of dozens of messages to welcome the participants to the group chat and encourage the participants to write to the group about any issues they had using the app. It was clear that in a few of the issues reported, the instructions simply needed to be made clearer at orientation (eg, the fact that multiplayer functionality was only enabled at that stage for the racing game); most of the other app-related issues were resolved by having a video chat to adjust the EMG thresholds during the first week of the study.

### User Perceptions—Quantitative Responses

The results from the questionnaire on the perceived usefulness of the WOW-Mobile app are presented in Table 5. Participants largely found the app to be useful. Participants reported that the app made it easy to track progress, increased motivation to exercise, and enabled participants to increase their level of physical activity. This information is presented in Table 5. Participants were also asked about the perceived ease of use of the WOW-Mobile app. Participants reported that the app was clear and easy to use. These data are presented in Table 6.

Finally, participants were asked about the usefulness of the various features of the app (Table 7). The highest rated game features were the single-player racing or spinning game and the boxing game. The lowest rated game was the resistance band game. The highest rated exercise features were the ability to monitor the heart rate and the ability to adjust EMG thresholds. The ability to monitor muscle activity was the lowest rated feature for all participants.

**Table 5:** Perceived usefulness of the app on a Likert scale ranging from 1 to 5 (higher scores indicate stronger agreement).

Perceived usefulness	Mean score (SD)
Using the mobile fitness app enabled me to increase my level of physical activity	4.25 (0.96)
Using the mobile fitness app enabled me to reach my peak fitness levels	3.75 (0.5)
Using the app improved my motivation to exercise	4.5 (1.0)
Using the app enhanced the effectiveness of a workout session	4.0 (0)
Using the app made it easier to track my progress	4.7 (0.77)
I found the app useful for improving, and then maintaining, fitness level	3.3 (0.5)

**Table 6:** Perceived usability of the app on a Likert scale ranging from 1 to 5 (higher scores indicate stronger agreement).

Perceived usability	Mean score (SD)
How to operate the app is clear	4.5 (5.8)
I found it easy to get the app to do what I want it to what I wanted it to do	4.0 (1.7)
It would be easy for me to become skillful at using the app	4.3 (1.2)
Overall, I found the app easy to use	4.3 (1.2)

**Table 7:** Usefulness of various app features (both games and exercise monitoring features; ranked on a Likert scale from 1 to 5, with higher scores indicating more usefulness).

Usefulness of app features	Mean score (SD)
Single-player racing or spinning game	4.0 (1.2)
Boxing game	4.0 (1.2)
Resistance band game (*Break-It-Like-Junior*)	3.5 (1.0)
Multiplayer racing game	4.5 (1.0)
Leaderboard (seeing your ranking and score among the other app users)	4.5 (1.0)
Ability to monitor muscle activity	3.0 (0)
Ability to monitor heart rate	4.0 (1.2)
Flexibility to adjust EMG^a^ (muscle activity) thresholds for each game and muscle	4.0 (1.2)

^a^EMG: electromyography.

### User Perceptions—Qualitative Responses

The questionnaire also included open-ended responses. The participants reported that the most positive aspects of the app included monitoring their progress and that it keeps track of how much time was spent in each session, helped them to “exercise in an animated and engaging way,” and motivated them to work out. The most negative aspects of the app were reported to be glitches, app crashing, and that *some of the games can be interpreted as being created for children not adults*. There was one response to the free-response question: “Overall, I believe like anything, the app could use improvement, maybe look more modern, and include more features or different exercises but the fact that someone is creating an exercising app for people who are wheelchair bound is simply amazing.”

## Discussion

Although mobile technology is being leveraged for fitness monitoring [38,39], and now includes exergaming [16,18], these apps are not tailored for individuals in wheelchairs. The exergaming apps that are available do not focus on upper limb exercises and are not equipped to track isometric contractions, as used in resistance exercises recommended for individuals in wheelchairs [38,39]. An exergaming PC app was very recently developed [40], but our app was distinct in its design to enable exergaming for individuals in wheelchairs in at least 2 ways: (1) exergames by Garcia-Hernandez et al [40] are on a PC platform, whereas WOW-Mobile was designed to maximize the flexibility of where and when this system could be used to help overcome barriers to exercise; and (2) the games developed in the study by Garcia-Hernandez et al [40] do not require sustained isometric strengthening contractions, such as resistance arm band exercises that are recommended for CRT; rather, their games require short bursts of muscle contraction. The WOW-Mobile system achieved its design objectives of increasing the likelihood of improving fitness levels and providing individuals in wheelchairs with the independence and flexibility to work out in the convenience of their own home by using body-worn sensors that communicate wirelessly with the WOW-Mobile phone app. The exergaming enabled the 3 of the 4 participants who had lower mobility impairment and had not previously exercised in the convenience of their own home to do so regularly (Figure 7 and Figure 9—S1, S3, and S4). Analysis of the participants’ EMG indicated that when users increased their time on the app, they burned more calories. SDs in minutes of exercising per week ranged from 14 to 61 minutes, which, based on participant feedback, was because of variability in busyness from week to week. Even after drops in exercise, participants still tended to resume more typical levels of exercise (Figure 9), indicating that they were incorporating exercise into their lifestyle, not just letting it be a one-time spurt of commitment. The participants with paraplegia who had good hand mobility had more consistent percent time exercising, whereas the one participant with tetraplegia showed a logarithmic rise in time efficiency on the app (Figure 9). This learning curve pattern indicates that it took time to settle into a routine and become accustomed to using the WOW-Mobile system, but by week 3, he already reached similar levels of efficiency as the rest of the cohort. According to feedback from the perceived usability and usefulness questionnaire as well as on the mobile messaging app, the app was usable and exercised more motivating.

On the basis of the problems that were expressed on the group chat, the most problematic issues using the app were due to lost internet connectivity, assigning sensors incorrectly, or difficulty setting appropriate EMG thresholds for each game to ensure that the users were challenged to reach an appropriate effort level. We are currently working on developing an offline version of the app that does not require continuous cloud server communication and devising algorithms to automate the threshold setting process. We have also been improving the user interface to make the assignment of sensors more user-friendly.

The mobile app, while allowing participants to perform holistic upper limb exercises, did not explicitly facilitate the circuit training prescribed by our team and others. When playing the exergames, the users were motivated to reach high scores and did not necessarily pace themselves as would be done in a circuit training program guided by knowledgeable physical trainers. One way to overcome this limitation is to design the games such that compliance with the desired workout is measured and users gain higher scores for closer compliance with the workout. For example, for the racing game, the physical trainers could create their own sessions exercising for the prescribed duration and intensity levels. The participants could select these sessions as the ghost player in a multiplayer game, and the objective of the game would be to remain within a certain distance from the ghost player. The WOW-Mobile fitness app already has the basic functionality built in to carry out such a protocol. Future versions of the app will include an option to play the games in a preset sequence rather than the user choosing games and number of repetitions.

The multiplayer gaming and leaderboard features were designed to make exercise and mobile fitness feel like an activity that could be done in a community with friends. One limitation of the study is that the participants did not have much opportunity to build community with each other before beginning to use the app, nor were they given opportunities to get to know each other outside of using the app together, and therefore, the potential of these features to help motivate users to play the games, and therefore exercise more, could not be evaluated in this study. Despite the participants not knowing each other before the study, the participants with thoracic level injuries strongly agreed that the app motivated them to exercise. We observed that for one participant who had impaired hand mobility, using the app would take substantially more effort and tedium to use, for example, to tap the screen to navigate the app. Although we can only speculate from our pilot study over differences in perception by age, gender, level of injury, and access to exercise facilities at baseline, the results provide us more basis for hypotheses to test in the future. For example, given that the one participant with a cervical level injury consistently rated the app’s usefulness lowest out of all the participants, we would hypothesize for future studies that WOW-Mobile is effective for individuals whose lower mobility is impaired but not their hand mobility. In addition, the participants who only rated the app’s usefulness with 4s and 5s did not exercise regularly before the study. This is consistent with our hypothesis that WOW-Mobile helps individuals with lower mobility impairment to overcome barriers to exercise.

On average, participants rated the usefulness of all the features between agree and strongly agree (4.0-4.5), with the exception of the monitoring EMG, for which the average was 3.75/5. They expressed valuing the ability to monitor their heart rate on the app, but less so on muscle activity. This could be due to target heart rates being more common knowledge and people, in general, being more accustomed to seeing heart rate. Therefore, we plan to design a more relatable metric based on EMG, such as calories burned, in future versions of WOW-Mobile.

The participants reported agreeing or strongly agreeing that it was clear how to use the app and that the app was easy to use but were more neutral on getting the app to do when they wanted it to do. The latter is consistent with the issues that were reported on WhatsApp, which were either already resolved or related to app functionality that is tied to network connectivity and the requirement to be connected to their user account on the cloud. We plan to develop an offline mode in which users can still enjoy, albeit limited, functionality even when the connection to the cloud drops.

The barriers to exercising that the participants were able to overcome by using the WOW-Mobile system included lack of access to adapted gyms, transportation to physical therapy clinics or gyms, and cost of physical therapy or gym memberships. Furthermore, anecdotal evidence indicates that being able to exercise in the comfort and privacy of one’s own home helped the participants overcome self-consciousness with their disability [41]. For example, one participant wanted help with a problem setting threshold but was reluctant to do a video chat; this same participant was the only one who declined a photo or media release form. Other participants expressed wanting to know what they looked like while exercising and expressed discomfort with having to be transferred while others besides the regular personal assistant were around. The fact that all these participants spent between 89 and 267 minutes per week carrying out their workout in wheelchairs for 8 weeks indicates that mobile app–based EMG-driven exergaming is a promising approach to facilitate and encourage regular exercise for individuals in wheelchairs.

### Conclusions

We have developed a mobile app–based fitness system that provides individuals in wheelchairs with a flexible way to carry out a goal-oriented, holistic workout with motivating feedback. We have bench tested the WOW-Mobile system and found the system to meet design specifications to support a circuit training workout that has been recommended for people with SCI and which supports individuals in wheelchairs to overcome existing barriers to regular exercise, including transportation and financial means to access gyms with adapted equipment and frequent and regular in-person visits with physical trainers. We also tested and verified the feasibility of individuals in wheelchairs using the WOW-Mobile system in their own homes. The participants who had thoracic level injuries and maintained hand mobility benefited most from WOW-Mobile and reported strong agreement with the overall ease of use of the app as well as the usefulness of the app in motivating them to exercise and enabling them to exercise more. As noted by one of the participants, mobile fitness tailored for individuals in wheelchairs is an unmet need and “the fact that someone is creating an exercising app for people who are wheelchair bound is simply amazing.”

## Appendix

Multimedia Appendix 1 Demonstration of connecting the wireless sensors.

Multimedia Appendix 2 Demonstration of the high striker or resistance arm band exergame.

Multimedia Appendix 3 Demonstration of the multiplayer racing or spinning exergame.

Multimedia Appendix 4 Demonstration of the boxing exergame.

## Abbreviations

CRT: circuit resistance training
EMG: electromyography
iEMG: integrated electromyography
MVC: maximum voluntary contraction
SCI: spinal cord injury
WOW: Workout on Wheels
